# Atherosclerosis and the Bidirectional Relationship Between Cancer and Cardiovascular Disease: From Bench to Bedside, Part 2 Management

**DOI:** 10.3390/ijms26010334

**Published:** 2025-01-02

**Authors:** Giuseppina Gallucci, Mario Larocca, Alessandro Navazio, Fabio Maria Turazza, Alessandro Inno, Maria Laura Canale, Stefano Oliva, Giulia Besutti, Andrea Tedeschi, Daniela Aschieri, Antonio Russo, Stefania Gori, Nicola Silvestris, Carmine Pinto, Luigi Tarantini

**Affiliations:** 1 Independent Researcher, 85025 Melfi, Italy; pina.gallucci@tiscali.it; 2 Provincial Medical Oncology, Department of Oncology and Advanced Technologies, AUSL—IRCCS in Tecnologie Avanzate e Modelli Assistenziali in Oncologia, 42100 Reggio Emilia, Italy; mario.larocca@ausl.re.it (M.L.); carmine.pinto@ausl.re.it (C.P.); 3 Cardiologia Ospedaliera, Department of Specialized Medicine, AUSL—IRCCS in Tecnologie Avanzate e Modelli Assistenziali in Oncologia, 42100 Reggio Emilia, Italy; alessandro.navazio@ausl.re.it; 4 Independent Researcher, 50100 Firenze, Italy; turazzafabio@gmail.com; 5 Oncologia Medica, IRCCS Ospedale Sacro Cuore Don Calabria, 37024 Negrar di Valpolicella, Italy; alessandro.inno@sacrocuore.it (A.I.);; 6 Division of Cardiology, Azienda USL Toscana Nord-Ovest, Versilia Hospital, 55041 Lido di Camaiore, Italy; marialaura.canale@uslnordovest.toscana.it; 7 UOSD Cardiologia di Interesse Oncologico IRCCS Istituto Tumori “Giovanni Paolo II”, 70124 Bari, Italy; s.oliva@oncologico.bari.it; 8 Radiology Unit, Department of Imaging and Laboratory Medicine, AUSL—IRCCS di Reggio Emilia, 42100 Reggio Emilia, Italy; giulia.besutti@ausl.re.it; 9 Department of Surgical and Medical Sciences of Children and Adults, University of Modena and Reggio Emilia, 41100 Modena, Italy; 10 Cardiology Unit of Emergency Department, Guglielmo da Saliceto Hospital, 29100 Piacenza, Italy; andrea.tedeschimd@gmail.com (A.T.); d.aschieri@ausl.pc.it (D.A.); 11 Department of Precision Medicine in Medical, Surgical and Critical Care (Me.Pre.C.C.), University of Palermo, 90127 Palermo, Italy; antonio.russo@usa.net; 12 Medical Oncology Department, IRCCS Istituto Tumori “Giovanni Paolo II”, 70124 Bari, Italy; silvestrisnicola@gmail.com

**Keywords:** atherosclerosis, cardiovascular disease, cancer, exposome, syndemic, preventome

## Abstract

The first part of this review highlighted the evolving landscape of atherosclerosis, noting emerging cardiometabolic risk factors, the growing impact of exposomes, and social determinants of health. The prominent role of atherosclerosis in the bidirectional relationship between cardiovascular disease and cancer was also discussed. In this second part, we examine the complex interplay between multimorbid cardio-oncologic patients, cardiometabolic risk factors, and the harmful environments that lend a “syndemic” nature to these chronic diseases. We summarize management strategies targeting disordered cardiometabolic factors to mitigate cardiovascular disease and explore molecular mechanisms enabling more tailored therapies. Importantly, we emphasize the early interception of atherosclerosis through multifactorial interventions that detect subclinical signs (via biomarkers and imaging) to treat modifiable risk factors and prevent clinical events. A concerted preventive effort—referred to by some as a “preventome”—is essential to reduce the burden of atherosclerosis-driven chronic diseases, shifting from mere chronic disease management to the proactive promotion of “chronic health”.

## 1. Introduction

The secular trend in hospital admissions indicates an increasing recognition of cancer among patients hospitalized for cardiovascular disease (CVD) [1]. Population-based studies further demonstrate that optimal control of atherosclerotic cardiovascular disease (ASCVD) risk factors—through appropriate behaviors and treatments, as recommended by the American Heart Association (AHA) [2]—reduces the incidence of both cancer and CVD and lowers all-cause, cardiovascular, and cancer-specific mortality [3,4]. These interventions include maintaining healthy sleep patterns, avoiding smoking, achieving normal plasma glucose and cholesterol levels, adopting a healthy diet, preventing obesity, engaging in regular physical activity, and managing blood pressure within the normal range. Importantly, these findings support the hypothesis of a shared pathophysiological “common soil” between cancer and CVD [5]. This notion is further emphasized by contemporary international clinical guidelines, which highlight cancer as a clinical condition warranting special attention in the management and prevention of ASCVD [6,7].

Unfortunately, ASCVD risk is often underestimated and undertreated in oncological patients [8,9,10,11,12,13] and the interactions between environmental exposures (the exposome) and cardiometabolic risk factors remain dangerously neglected. This situation is no longer acceptable, especially given the progressive improvement in cancer patient survival [14,15] due to advancements in oncological diagnosis and therapy, the aging of the general population [16,17], and the increasing incidence of early-onset cancer (before the age of 50). In the first part of this review, we discussed the evolving landscape of atherosclerosis and the central role of chronic low-grade inflammation in both CVD and cancer [18]. In this second part, we will explore the intricate interconnectedness of environmental exposures and cardiometabolic risk factors through a syndemic approach, which adds complexity to the management of contemporary oncological patients. From this perspective, we aim to define an updated strategy for managing ASCVD in patients with a history of or active cancer, with a particular focus on preventive cardio-oncology.

## 2. The Complexity of Current Oncologic Patients

Currently, at least 80% of heart disease, stroke, and type 2 diabetes, as well as 40–50% of cancers, are linked to an unhealthy lifestyle [19,20,21,22,23]. Cancer patients often present with a high cardiovascular (CV) risk or a history of heart disease, frequently associated with exposure to multiple risk factors, which often cluster together [24,25,26,27]. The CV risk includes both the direct and indirect CV effects of cancer and oncologic treatments, impacting not only physical health but also mental well-being [28,29].

There is a growing population of long-term cancer survivors requiring vigilant and continuous surveillance to detect disease recurrence, CV complications, or second cancers. Some of these patients are living with advanced metastatic cancer, maintained in remission through prolonged and ongoing treatments [30]. In this scenario, patient management must also address socioeconomic factors associated with cancer treatment. The improved survival rates and “chronicity” of cancer, facilitated by expensive therapies and continuous medical investigations, give rise to the issue of “financial toxicity”—the economic strain linked to the long-term care necessary to maintain favorable outcomes and prevent complications, particularly when cancer and CVD coexist in the same patient [31,32,33,34,35,36]. Financial toxicity, encompassing both the objective financial burden and the subjective distress caused by a cancer diagnosis and its treatment, has significant implications not only for healthcare systems but also for the quality of life of cancer patients and their families. As highlighted by the ESMO expert consensus statements, socioeconomic determinants play a critical role in this regard [37]. They include “intrinsic” factors (e.g., gender, age, ethnicity, and lower income), “disease-related” factors (e.g., costs of systemic anticancer therapies), and “extrinsic” factors (e.g., travel expenses, out-of-pocket healthcare costs, lost wages, and medical appointments or tests) [38,39,40,41]. In addition, comorbidities have a relevant impact: in a cohort of long-term survivors of adolescent and young adult (AYA) cancer from the Kaiser Permanente database, 40% of patients had multiple comorbidities 10 years after diagnosis. The most common atherosclerosis-related comorbidities included dyslipidemia (22 per 1000 person-years), hypertension (16 per 1000 person-years), diabetes (10 per 1000 person-years), and severe depression or anxiety [42]. More recently, in the St. Jude Lifetime Cohort of childhood cancer survivors [43], 45.5% of individuals aged 40–49 had prediabetes and 14% had diabetes. Over a median follow-up of 5.1 years, 10% of those with prediabetes progressed to diabetes. Survivors with a worse cardio-metabolic profile experienced significantly more CV events, including myocardial infarction (MI) in those with prediabetes and cardiomyopathy or stroke in diabetics.

Preventing and managing the risk of ASCVD is even more important in patients with adult-onset cancer. Aging, often accompanied by chronic low-grade inflammation (inflammaging), is a major risk factor for CVD and cancer [44,45]. Currently, more than 50% of elderly cancer patients live with two or more chronic conditions, with CVD, diabetes, lung disease, and chronic kidney disease (CKD) being the most prevalent; these conditions may worsen during and after cancer treatment [46,47,48,49,50,51,52,53,54,55]. The clinical complexity in these cases is often compounded by hazardous environments, further complicating the management of both cancer and comorbidities [56]. The exposome concept, which contextualizes patients within their environment and lifestyle, encompasses biochemical and metabolic changes resulting from various lifelong exposures; these components, as identified by Münzel [57,58], play crucial roles in the development of both CVD and cancer. Moreover, the interaction between exposomes and metabolic disorders is not merely additive but represents a “syndemic”, a term introduced in 1996 to describe an “interrelated complex of health and social crises” [59].

According to harmonized data from 112 cohort studies involving 1,518,028 participants (54.1% women) across 34 countries and eight geographic regions, with a median age of 54.4 years and a median follow-up of 7.3 years, approximately 57.2% of incident CVD in women and 52.6% in men, as well as 22.2% and 19.1% of deaths from any cause, respectively, were attributable to five modifiable risk factors: body mass index (BMI), systolic blood pressure, non-high-density lipoprotein cholesterol (non-HDL-C), current smoking, and diabetes [23]. The Global Burden of Diseases, Injuries, and Risk Factors Study (GBD) 2019 further emphasizes that a large proportion of cancer deaths are linked to modifiable risk factors, with smoking, alcohol use, and high BMI contributing the most. Additionally, metabolic risk factors saw the largest increase between 2010 and 2019 (34.7%) [21].

The rise in obesity and related conditions, particularly metabolic syndrome (MetS), is unsurprising given the increasing prevalence of energy-dense food consumption, sedentary behavior, urbanization, and lower socioeconomic status [60,61,62]. Obesity is commonly associated with hypertension, dyslipidemia, accelerated atherosclerosis progression, and increased risk of several cancers including colorectal, gallbladder, pancreatic, kidney, liver, and breast cancers (in post-menopausal women) [60,63,64,65]. Alarmingly, the incidence of obesity has risen sharply among children and adolescents. Overweight or obesity in childhood is linked to future cancer, type 2 diabetes, and ASCVD, often before the age of 50 [66,67,68,69,70]. In young patients with “obesity-related” cancer, ASCVD risk should not be underestimated, as cardiometabolic risk factors often emerge during childhood and adolescence [71,72]. For example, the PESA (Progression of Early Subclinical Atherosclerosis) study, which examined the vascular systems of 4184 asymptomatic individuals aged 40–54, found that over 60% (71% of men and 48% of women) had silent atherosclerosis in one or more vascular regions [73] with progression observed in over 40% after a 3-year follow-up [74].

In recent years, there has been increasing focus on refining traditional CV risk factors using indices derived from common lab tests. One promising parameter is the TyG index, calculated as the logarithmized semi-product of fasting levels of triglycerides and glucose: ln [triglycerides (mg/dL) × blood glucose (mg/dL)/2]. The TyG index is highly sensitive and specific for insulin resistance [75,76], correlates with metabolic syndrome [77], and is a predictor of type 2 diabetes and CVD events [78,79], particularly in low- and middle-income countries [80]. Additionally, the TyG index has been associated with cancers of the kidney, liver, pancreas, and colorectum and mediates a significant portion of the effect of BMI on cancer risk [81,82]. Recent meta-analyses, finally, indicate that the Tyg index is a proxy for coronary artery disease (CAD) risk prediction, severity assessment, and prognosis evaluation [83,84].

Despite its simplicity and affordability, the TyG index has limitations, as most studies are based on single measurements and lack data on confounding factors such as diet, physical activity, and alcohol intake. Furthermore, insights regarding demographic categories such as children, adolescents, and women remain at a preliminary stage [85]. Prognostic accuracy may be enhanced by incorporating inflammatory markers such as high-sensitivity C-reactive protein (hsCRP) or cell-derived markers such as neutrophil–lymphocyte ratio (NLR) [86,87,88,89,90,91,92,93,94]. Another important risk factor is non-HDL-C, particularly triglyceride-rich lipoproteins, which have stronger athero-genic potential than low-density lipoprotein cholesterol (LDL-C) [95].

Addressing obesity requires a focus not just on the quantity of adipose tissue but also on its type (brown vs. white adipose tissue) and distribution [96,97]. Advanced body composition methods, such as dual-energy X-ray absorptiometry (DEXA) and computed tomography (CT), have shown the limitations of BMI in predicting CV risk, especially in oncologic patients with normal or reduced BMI [98,99,100,101]. These fat deposits, including intramuscular and pericardial fat, contribute to systemic inflammation and accelerated atherosclerosis. “Sarcopenic obesity”, characterized by reduced muscle mass and strength alongside fat accumulation, is common in cancer patients [102] and is linked to subclinical atherosclerosis and CVD events [96,97,103,104,105,106,107]. Chronic inflammation plays a major role in sarcopenia, with hsCRP and NLR serving as useful markers for silent atherosclerosis [108,109,110,111]. This is particularly relevant for patients on immune checkpoint inhibitors (ICIs); preliminary observations identify sarcopenia and high hsCRP as independent risk factors for poor outcomes in cancer patients treated with ICIs [112,113,114].

As we have pointed out, inadequate treatment of CV risk factors is common in oncology patients and may worsen cancer prognosis while increasing the likelihood of CV events. In the prospective multicenter RADICAL-PC program, which involved newly diagnosed prostate cancer (PCa) patients scheduled for androgen deprivation therapy (ADT), obesity, hypertension, and hypercholesterolemia were the most common comorbidities, yet they were often inadequately managed [8]. Similar findings were reported by Sun in a retrospective analysis of PCa patients from the US Veterans Database [9]. Additionally, the CARDIOTOX Registry revealed that hypercholesterolemia and elevated blood pressure were frequently overlooked and poorly controlled [10]. A recent study by Lin et al. demonstrated that pretreatment lipid profiles may contribute to resistance to ADT with androgen receptor pathway inhibitors in localized cancer, based on findings from both human cohorts and explant models [115]. Likewise, non-adherence to Life’s Simple 7 [116] was associated with increased risk of ASCVD (MI, angina, stroke, and heart failure) in long-term survivors of gastric, colorectal, and breast cancers enrolled in the Japanese National Registry [4].

On the contrary, adherence to a healthy lifestyle, as recommended by guidelines, has been shown to reduce the risk of both cancer and ASCVD in the general population, as well as in high-risk groups such as individuals with high polygenic risk [117], diabetes [118], or CKD [119]. Emerging evidence suggests that these benefits may also extend to cardio-oncology patients [3].

Hypertension, the most common risk factor for ASCVD, is a leading comorbidity in cancer patients at all stages—before, during, and after treatment [23]. Many oncologic therapies can induce de novo hypertension or worsen pre-existing high blood pressure [120] through several mechanisms, including oxidative stress, endothelin-1 activity, prostaglandin imbalance, endothelial dysfunction, increased sympathetic activity, microvascular rarefaction, and reduced nitric oxide production [121,122]. Managing hypertension during cancer treatment is challenging, especially as it is often accompanied by a worsening cardiometabolic risk profile, such as during hormonal therapy for breast or prostate cancer [123,124,125]. In a large retrospective cohort of cancer survivors, maintaining blood pressure within normal ranges (<130/90 mmHg) per international guidelines was associated with a reduced incidence of CV complications, particularly in patients with elevated cardiometabolic risk [126].

Although an individual’s absolute CV risk is key to guiding treatment, especially in primary prevention, risk prediction models have evolved in recent years to reflect the growing prevalence of cardiovascular–kidney–metabolic (CKM) conditions, such as obesity, diabetes, and CKD, often clustering with adverse social factors like living in socioeconomically deprived neighborhoods [127,128,129]. In Europe, the Systematic Coronary Risk Estimation 2 (SCORE2) and SCORE2-Older Persons (SCORE2-OP) are widely used [6]. In the U.S., the AHA’s recent PREVENT equations have been validated to predict risk in adults aged 30–79 without known CVD [130]. PREVENT uses traditional risk factors (e.g., smoking, systolic blood pressure, cholesterol, and diabetes) and estimated glomerular filtration rate, with models being sex-specific, race-neutral, age-scaled, and adjusted for non-CVD death [130]. However, none of these CV risk scores consider the unique challenges of cancer patients, including the elevated CV risk due to shared risk factors, the CV toxicity of cancer therapies, and cancer itself [131]. As recent research from the UK Biobank shows, established CV risk scores such as QRISK3, SCORE2, Framingham, and others do not perform as well in cancer survivors compared to non-cancer patients [13]. To address these limitations, coronary imaging—such as coronary artery calcium (CAC) scoring and coronary computed tomography angiography (CCTA)—can enhance the prognostic accuracy of clinical scores [132,133]. CCTA has shown promise in detecting coronary inflammation through radiological changes in perivascular adipose tissue (PVAT), measured via the Fat Attenuation Index (FAI) [134]. An oncology-like screening approach for CVD may be particularly effective to identify preclinical signs of atherosclerosis, or “the lump in our artery” [133], and this can be achieved via meticulous evaluation of routine tests used in cancer patients such as computed tomography (CT) that can detect vascular calcifications. Advanced vascular imaging, especially if artificial intelligence-assisted, offers near-histologic evaluation of plaque composition and progression; positron emission tomography (PET) imaging can reveal molecular and cellular events before structural changes arise; cardiac CT provides a complete assessment of fractional flow reserve, perfusion, peri-coronary adipose tissue, plaque characterization, and cardiac abnormalities; and hybrid PET-CT imaging allows simultaneous evaluation of cardiac perfusion and coronary anatomy in a single scan. In addition, inflammation biomarkers, such as hsCRP and interleukin-6 (IL-6), have long been associated with CV risk independent of cholesterol levels. Numerous trials demonstrate improved outcomes with reductions in both LDL-C and CRP [135,136,137,138,139,140,141]. A recent meta-analysis of 53 randomized controlled trials (RCTs) involving 171,668 participants showed significant reductions in CRP levels from lipid-lowering therapies such as statins, bempedoic acid, ezetimibe, and omega-3 fatty acids, independent of LDL-C reduction [142]. Notably, hsCRP predicted future CV events more effectively than LDL-C in high-risk, statin-intolerant patients [143].

## 3. How to Manage Atherosclerosis-Driven Chronic Diseases in Cancer Patients

The first step in management is prevention. The complexity of cancer patients with their syndemics makes prevention a difficult task for cardio-oncologists. At baseline, cardiovascular health (CVH) should be optimized and risk factors must be actively searched for and managed earlier, more intensively, and with greater precision, adopting the principles widely used in cardiology: “the earlier, the better” and “strike early, strike strong” [144]. Machine learning-based recalibration of CV risk models, as proposed by Zinzuwadia et al. [145], may further improve risk stratification within local healthcare systems. This vigilant approach should be sustained during and after cancer treatment to detect early signs of cardiotoxicity, thus allowing timely interventions and prevention of clinical events.

### 3.1. NON-Pharmacologic Treatments

Risk factors such as tobacco use; unhealthy diets high in saturated and trans fats, salt, and sugar; physical inactivity; and excessive alcohol consumption are responsible for more than two-thirds of all new NCD cases and significantly increase the risk of complications [23].

Diet. Meta-analyses of observational studies and randomized trials confirm the protective effect of Mediterranean diet against all-cause mortality, CVD, CAD, MI, cancer, neurodegenerative diseases, and diabetes [146,147]. A strong inverse association between Mediterranean diet adherence and cancer risk has also been observed, particularly for breast cancer (BC) [148]. The MOLI-SANI study further underscored the importance of dietary quality, demonstrating that higher olive oil consumption is linked to lower rates of cancer, CV, and all-cause mortality, independent of overall diet quality [149].

In contrast, there is growing concern about the health impact of ultra-processed foods (UPFs), which are typically high in energy, sugars, unhealthy fats, and salt, and low in fibers, protein, vitamins, and minerals. A systematic review of eight retrospective and three prospective cohort studies found that a 10% increase in UPF daily consumption was associated with a higher risk of overall cancer (HR = 1.13, 95% CI 1.07–1.18) [150]; in children, higher UPF intake is significantly associated with increased adiposity and worse cardiometabolic risk [151]. Reducing UPF consumption is crucial, as outlined by guidelines [131,152].

Microbiota and microbiome. Dysregulated microbiota can exacerbate inflammatory pathways, including IL-6, CRP, lipopolysaccharide (LPS), short-chain fatty acids (SCFAs), mitogen-activated protein kinase (MAPK), nuclear factor-kB (NF-kB), and oncometabolite production [153]. Emerging data suggest a link between microbiota and cancer development, as well as responses to oncological treatments [154]. Many bacterial species within the microbiome may play oncogenic roles, making the influence of bacterial flora potentially relevant in the context of personalized oncology treatments [155], and intestinal flora may impact the development of early-onset cancer [156,157,158] and the response to immunotherapy [159,160,161].

Alcohol. Alcohol consumption has been causally linked to several types of cancer, with no safe level of intake. The risk of alcohol-associated cancer increases with consumption and is consistent across all ethanol-containing beverages. The American Cancer Society’s Guidelines for Diet and Physical Activity for Cancer Prevention advise against alcohol consumption, stating that individuals who choose to drink should limit their intake to no more than one drink per day for women and two drinks per day for men [152].

Physical activity (PA) PA significantly impacts cellular processes and tumor growth [162] by modulating insulin and glucose metabolism, immune function, inflammation, sex hormones, oxidative stress, genomic instability, and myokines [152,163]. Furthermore, PA may reduce cancer risk related to obesity. The physical effects of PA, such as increased blood flow, shear stress on the vascular system, pH regulation, heat production, and sympathetic activation, along with endocrine effects like stress hormones, myokines, and circulating exosomes, may help regulate cancer progression and biology by affecting tumor growth, metastatic potential, tumor metabolism, and the immunogenic profile of tumors. Exercise also has beneficial effects on cancer symptoms and treatment-related adverse effects [164].

Smoking cessation is a critical priority for preventing both CVD and cancer. In 1964, the Surgeon General’s report first established the causal link between smoking and lung cancer [165]. Fifty years later, the 2014 report highlighted the increased risk of smoking exposure for several other cancers. While lung, laryngeal, and pharyngeal cancers have the highest relative risks for current smokers, elevated risks have also been observed for cancers of the upper digestive tract, oral cavity, lower urinary tract, esophagus, nasopharynx, cervix, pancreas, stomach, kidney, liver, and colorectal regions [166,167]. Meta-analyses have confirmed smoking as a major risk factor for urothelial bladder cancer [168,169] and for an unfavorable course of prostate cancer [170].

Social and psychological determinants of health. Low socioeconomic status is a recognized risk factor for many mental and behavioral problems that eventually lead to lifelong physical diseases [171,172]. Food insecurity (FI), or “the lack of consistent access to enough food for an active and healthy life” [173], has indeed been associated with ASCVD risk through the nutrition/anthropometric, psychological/mental health, and access to care pathways [174]. In 2019, Tawakol et al. documented a link between socioeconomic disparities and CVD through a stress-related neurobiological pathway [175]. In 2022, the American Heart Association (AHA) included sleep as an essential component of CVH and recognized the importance of social determinants of health (SDOH) and psychological well-being to achieve equitable CVH outcomes [2]. Positive psychological traits, such as optimism, purpose in life, environmental mastery, perceived social role reward, and resilient coping, have been associated with better CVH, while psychosocial stress and depression are linked to worse outcomes [2,176]. Loneliness, a recognized SDOH, is also a risk factor for both CVD and cancer [177,178,179]. In a systematic review of 51 cohort studies involving 2,611,907 participants with an average follow-up period of 10.3 years, depression and anxiety were associated with a significantly higher risk of cancer incidence (adjusted RR: 1.13; 95% CI: 1.06–1.19), specific cancer mortality (1.21; 1.16–1.26), and all-cause mortality in cancer patients (1.24; 1.13–1.35) [180]. Psychological distress is common in cancer [181,182] and may intersect with the cognitive change associated with therapy or “chemo-brain” [183], an entity that has been identified in people with a variety of cancers who have undergone chemotherapy and/or hormone treatment. These patients may have difficulties in executive functions, multitasking, short-term memory and attention, and coping ability. Anxiety and depression are also emerging as significant risk factors for subclinical atherosclerosis [184,185,186]; in cardio-oncology, there is an urgent need to address these novel risk enhancers. An example is offered by the positive impact of spirituality in serious health conditions and its ability to counter loneliness, hopelessness, and depersonalization [187]; to enhance coping abilities [188]; and to potentially improve outcomes through effects on the autonomic nervous system and hormonal, immunological, and neurological pathways [189]. In 2008, The Institute of Medicine (IOM) stated the following: “Attending psychosocial needs should be an integral part of quality cancer care. All components of the healthcare system involved in cancer patient management should explicitly incorporate attention to psychosocial needs into their policies, practices, and standards addressing clinical care” [190,191]. Psychotherapeutic interventions useful for mental health in cancer patients are based on emotional support and include education, behavioral training, group interventions, and individual psychotherapy.

Pollution, an increasingly significant health issue, acts as a “syndemic” when combined with traditional risk factors. Air and noise pollution, light disruption at night, climate change, and chemical exposure contribute to the development and progression of cardiometabolic multimorbidity [192]. Studies on populations living near airports, heavy road traffic, and industrial zones highlight the impact of environmental pollution on cardio-metabolic and oncological risks [57,193,194,195]. Addressing pollution from an “exposomic” perspective, which considers the total environmental exposure across an individual’s life, is a crucial issue.

### 3.2. Pharmacologic Therapies: Cardiometabolic and Anti-Inflammatory Agents

#### 3.2.1. Cardiometabolic Therapies and Their Potential Effect in Cardio-Oncology

The shared mechanisms between CVD and cancer explain the unexpected anticancer effects of many cardiometabolic drugs, largely due to metabolic remodeling in cancer cells.

1. Statins. Statins reduce low-density lipoprotein cholesterol (LDL-C) in a dose-dependent manner, lower triglycerides (TG) by 10–20% (with greater reductions from high-intensity statins), and have a minimal effect on lipoprotein(a) [Lp(a)] [196]. Their beneficial impact on CV morbidity and mortality is well-documented [197,198,199,200]. Statins may have anticancer properties due to their cholesterol-lowering effects [201,202] and ability to enhance the efficacy of ICIs [203,204]. Preclinical studies have shown statins’ direct antiproliferative and immunomodulatory effects, promoting immunogenic cell death in KRAS (Kirsten rat sarcoma viral oncogene homolog)—mutated cancer cells, through increased expression of “eat me” signals and damage-associated molecular patterns, while reducing proteins that suppress T cell antitumor responses [205].
2. PCSK9 inhibitors (PCSK9Is). PCSK9Is target the PCSK9 protein, which regulates LDL receptors (LDLRs). These inhibitors work through monoclonal antibodies (mAbs) that lower plasma PCSK9 levels by reducing its binding to LDLRs, and through a small interfering RNA (siRNA), Inclisiran, which inhibits PCSK9 synthesis. Statins increase circulating PCSK9 levels, enhancing the benefits of the mAbs. These drugs are recommended for secondary prevention in patients who do not achieve LDL-C targets with statins and ezetimibe [6,206]. Beyond LDL-C lowering, PCSK9Is have pleiotropic effects, such as reducing platelet reactivity, decreasing smooth muscle cell proliferation, limiting macrophage accumulation, and promoting plaque regression and stabilization [207]. Emerging data suggest a link between PCSK9 and cancer: PCSK9 gain-of-function variants are associated with higher LDL-C and an increased risk of BC, while loss-of-function variants show the opposite effect [208]. PCSK9Is may also enhance the anticancer efficacy of immune checkpoint inhibitors (ICIs), as seen in colorectal cancer, where PCSK9 inhibition boosts the effectiveness of PD-1 blockade by reducing LDL-R and transforming growth factor-β (TGF-β) levels [209].
3. Metformin. Untreated patients with type 2 diabetes (T2DM) have an increased risk of cancer, likely due to the growth-promoting effects of chronically elevated glucose and insulin levels. This heightened risk is most pronounced for cancers of the liver, pancreas, endometrium, colon, breast, and bladder [210]. The anticancer potential of metformin has been widely studied, but its preventive role remains debated. A recent review by Galal et al. [211] highlights metformin influence on cancer cell biology through its effects on energy metabolism, cellular growth, angiogenesis, and programmed cell death. Metformin exerts both direct (insulin-independent) and indirect (insulin-dependent) effects on cancer cells, which may interact with each other. However, in recent clinical trials, metformin failed to improve the clinical course of prostate cancer [212,213] and BC [214]. Metformin has also been proposed as an immuno-metabolic adjuvant for cancer therapy. Preclinical studies suggest that it can alter the tumor immune microenvironment [215,216] and reduce programmed death ligand 1 (PD-L1) expression, enhancing its degradation [217,218]. However, the “boosting” effect of metformin on cancer immunotherapy has been questioned due to confounding factors [219]. A meta-analysis of 22 studies involving over 9000 patients revealed a significant association between metformin use and poorer overall survival, suggesting an adverse prognosis when combined with immune checkpoint inhibitors (ICIs) [220]. Further research is needed to fully assess metformin’s clinical and immunomodulatory potential in cancer treatment.
4. Glucagon-like peptide 1 Receptor Agonists (GLP-1 RAs). GLP-1 RAs are incretin-based therapies that enhance insulin secretion in response to meals [221]. In addition to their role in glucose regulation, they promote weight loss, reduce chylomicron secretion, and lower blood pressure [222,223]. A meta-analysis of 40 randomized controlled trials demonstrated that GLP-1 RAs significantly reduce inflammatory markers such as CRP, tumor necrosis factor-alpha (TNF-α), and malondialdehyde (MDA) [224]. Liraglutide and Semaglutide have also been approved for obesity treatment after the SCALE and the STEP programs [225,226,227,228]. GLP-1 RAs may have a potential role in reducing obesity-related cancer risk [229]. A recent study involving over 1.6 million patients with type 2 diabetes (T2DM) compared the incidence of 13 obesity-associated cancers among those treated with GLP-1 RAs, insulin, or metformin. GLP-1 RA treatment was associated with a significant reduction in the risk of 10 cancers, including esophageal, colorectal, endometrial, gallbladder, kidney, liver, ovarian, and pancreatic cancers, as well as meningioma and multiple myeloma, compared to insulin-treated patients. When compared to metformin, GLP-1 RAs showed a beneficial effect in reducing the risk of colorectal and gallbladder cancers [230]. In preclinical studies, liraglutide has shown potential to enhance the anti-tumor efficacy of immune checkpoint inhibitors (ICIs) in lung and liver cancers [231]. However, conflicting data exist regarding the effects of GLP-1 RAs on cancer initiation and progression. The expression of the GLP-1 receptor (GLP-1R) varies across different tumor types, as well as between healthy and diseased tissues [232]. GLP-1R is highly expressed in endocrine tumors and has also been detected in embryonic cancers, nervous system tumors, and certain carcinomas. It is also expressed in various healthy tissues, including the pancreas, digestive tract, heart, skeletal muscle, liver, central nervous system, and immune cells, where it can be activated [222]. The effects of GLP-1 RAs appear to be tumor-specific. Notably, Semaglutide carries a boxed warning from the Food and Drug Administration (FDA) regarding the potential risk of thyroid C-cell cancer, specifically medullary thyroid carcinoma [233]; this increased risk has not been observed with liraglutide. A recent French multicenter registry reported an association between GLP-1 RA use for 1–3 years and an increased risk of thyroid cancers (adjusted hazard ratio [HR] 1.58, 95% CI 1.27–1.95) [234], a finding supported by a systematic review of 64 randomized controlled trials [235].
5. Sodium-Glucose Co-Transporter 2 (SGLT2) Inhibitors. According to the 2023 ESC Guidelines for managing CVD in patients with diabetes, empagliflozin, canagliflozin, dapagliflozin, and sotagliflozin are recommended for patients with T2DM and ASCVD to reduce CV events, regardless of baseline or target HbA1c levels and independent of other glucose-lowering treatments [236]. SGLT2 inhibitors have demonstrated antiproliferative effects against certain tumor types. The antineoplastic activity of SGLT2 Inhibitors is partially attributed to their ability to block glucose uptake in metabolically reprogrammed cancer cells expressing SGLT2 receptors. However, preclinical studies suggest that the anticancer effects of SGLT2 Inhibitors are multifactorial, involving several metabolic pathways [237]. Beyond their potential direct anticancer effects, SGLT2 Inhibitors may offer protective benefits against cancer therapy-induced CV toxicity. Preclinical studies have shown significant cardioprotective effects against anthracycline exposure [238,239] and ponatinib-induced cardiac toxicity [240]. Clinical studies have also reported favorable outcomes in patients with cancer and T2DM treated with anthracyclines [241], as well as improved outcomes in patients with cancer therapy-related cardiac dysfunction or heart failure [242].

#### 3.2.2. Anti-Inflammatory Agents in CVD and Cancer

The critical role of inflammation in atherosclerosis has sparked interest in clinical trials investigating anti-inflammatory therapies, such as the Canakinumab Anti-inflammatory Thrombosis Outcome Study (CANTOS) with the IL-1 inhibitor canakinumab [243] and colchicine studies [244,245,246] with potential interest in cardio-oncology.

Interleukin inhibitors. The contradictory impact of selective inhibition of IL-1β observed with canakinumab on non-small cell lung cancer [247,248,249,250] highlights the complex, pleiotropic role of IL-1 signaling [251]. Elevated IL-1 levels are associated with poor prognosis in various cancers [252] and promote carcinogenesis by driving chronic inflammation and establishing a protumor cytokine network [253]. Furthermore, IL-1 exerts paradoxical effects on antitumor immunity. While it enhances the activation of natural killer- and T-cells, promoting antitumor activity [254], it also contributes to immunosuppression by facilitating the expansion and mobilization of immune cells such as myeloid-derived suppressor cells (MDSCs) [255]. As a result, therapeutic strategies targeting IL-1 require further clinical studies to determine the efficacy and optimal use of anti-IL-1 therapies in specific clinical contexts. Theoretically more promising is the IL-6 block [256], Ziltivekimab, a monoclonal antibody targeting the IL-6 ligand that significantly reduces inflammation and thrombosis biomarkers linked to atherosclerosis. Furthermore, IL-6 plays a key role in tumorigenesis, cancer progression, and treatment resistance [257]. In preclinical studies, IL-6 inhibition combined with immune checkpoint blockade (ICB) enhanced antitumor immunity and slowed tumor progression across various cancer models [258,259,260]. IL-6 is also implicated in the development of immune-related adverse events (irAEs) associated with ICB, as shown by the effectiveness of tocilizumab, an IL-6 receptor inhibitor, in managing these events in clinical practice. Thus, combining IL-6 inhibitors with ICB holds promise for improving cancer immunotherapy while reducing the risk of adverse events [261,262], including atherosclerosis [263]. However, more definitive data are expected from the ongoing trials [264].

Colchicine is a potent anti-inflammatory agent that works by inhibiting microtubule polymerization, neutrophil extracellular trap (NET) release, platelet activation, and the nucleotide oligomerization domain-like receptor protein 3 (NLRP3) inflammasome [265,266]. Its effects on the NLRP3 inflammasome limit the activation of inflammatory cytokines, such as interleukin-1 and interleukin-18, in response to danger signals. Colchicine reduces hsCRP levels and may decrease coronary artery plaque volume [267]. A low-dose regimen (0.5 mg daily) has been approved by the FDA for secondary prevention in patients with CAD [268]. The drug’s role in oncogenicity remains unclear. A study of 85,374 Israeli patients with Familial Mediterranean Fever (FMF) showed a significantly lower incidence of cancer compared to the general population, potentially due to colchicine treatment [269]. Shared risk factors between gout and cancer (e.g., obesity and alcohol) have suggested a potential cancer susceptibility in gout patients, as demonstrated in a study of 8408 male gout patients [270]. A further analysis of 24,050 gout patients found that those diagnosed with cancer were older and had a lower rate of colchicine prescriptions than those without cancer [271] Interestingly, preclinical studies have shown a cardioprotective effect of low-dose colchicine in doxorubicin-induced cardiotoxicity, likely through the restoration of autophagy [272]. A recent study has documented, in mice and humans carrying clonal hematopoiesis of indeterminate potential (CHIP)-mutations, that colchicine can blunt the higher risk of ASCVD associated with somatic TET2 mutation-driven CHIP by suppressing IL-1β overproduction [273].

Table 1 illustrates the complexity of the “preventome”, a multifactorial intervention that requires the commitments of individuals and communities to defeat the “exposome”, the sum of many different exposures (e.g., CV and cancer risk factors deriving from an unhealthy lifestyle, and psychological and environmental issues).

## 4. Conclusions

**Figure 1:**
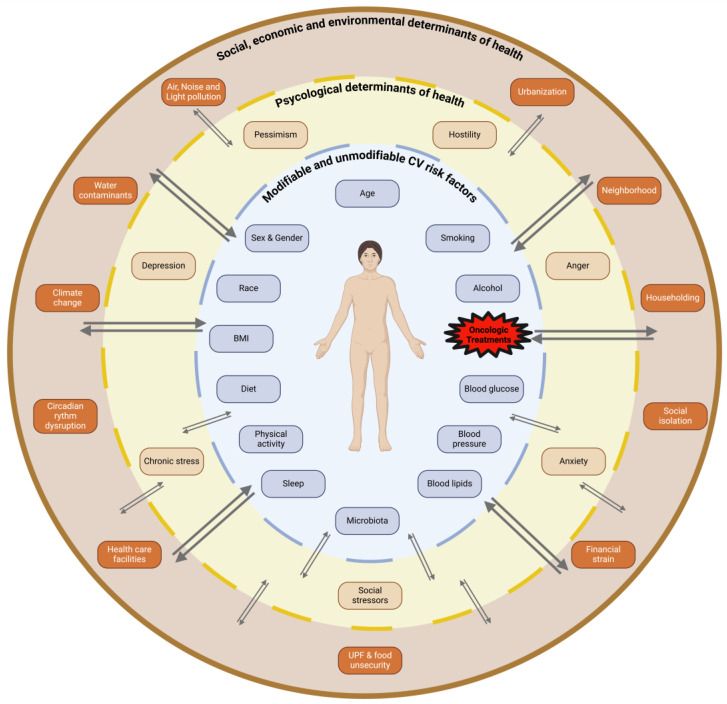
The figure illustrates the cardio-oncology patient as a target of many syndemics: in the inner circle, the clustering of cardiovascular risk factors and the effects of the oncologic treatments; in the first outer circle, the psychological determinant of health; and in the external circle, the social determinants of health including environmental exposomes. The arrows illustrate how the various health conditions cluster and interact and how harmful alignments can lead to dangerous conditions mimicking the “Swiss Cheese” effect [276].

The evolving landscape of atherosclerosis—shaped by emerging exposomes, cardiometabolic, socioeconomic, and psychological factors—and the complex role of immunity with a growing interest in complement (the so-called immuno-atherosclerosis) [274,275], has heightened the complexity of cardiac patient management. In this scenario, cardio-oncology patients present unique challenges due to the overlap of CVD and cancer, compounded by the cardiotoxic effects of cancer treatments. As we have seen, prevention is therefore essential and it has to start at baseline, with a comprehensive evaluation of all the complexities: the modifiable risk factors (the essential 8), the unmodifiable risk factors (e.g., age), the psychological determinants of health, and the socioeconomic background. As illustrated in Figure 1, all of the risk factors in the inner circle (the CVH components and the effects of the oncologic treatments) may interact with the components of the other circles and all the interactions have a syndemic nuance. The patient may be the target of all these syndemic effects, and only a proactive multidisciplinary intervention can prevent the dangerous “Swiss cheese effect” [276].

Tackling the root social and environmental causes of metabolic disorders should be a priority. Seen from this perspective, cardio-oncology serves as a valuable tool for identifying novel disease mechanisms, following a unique pathway from clinical observations through preclinical functional studies to clinical validation and drug development, as recently highlighted by Moslehi [277]. Addressing inequity in preventive measures is also urgent; policymakers and communities must support cardio-oncology teams in expanding equitable access to essential healthcare resources.

## Figures and Tables

**Table 1:** The “PREVENTOME”: how to manage CV and cancer risk factors, who is in charge, the pathways, and the desirable targets.

Life style	**Risk Factors**	**Committed Subjects**	**Non-Pharmacological** **Management**	**Pharmacological** **Management**	**Involved** **Pathways**	**Targets**
	Obesity	Patient, GP, caregiver	Healthy diet, exercise	GLP-1 RAs	CRP, TNF-α, MDA	Optimal BMI [2,6], reduction in obesity-related cancer [152]
	Hypertension	Patient, GP, caregiver	Diet (Restriction of sodium to ~2 g per day, MD, DASH, moderate or no alcohol), exercise	ACE-Is, ARBs, CCBs	Impaired NO production, oxidative stress, and endothelial dysfunction	Tailored to CV risk, comorbidities, and risk modifiers [6]
	Diabetes	Patient, GP, caregiver	Diet, exercise	Metformin, GLP-1 RAs, SGLT2-Is	AMPK, IGFR, mTORC1, ETC, β-Catenin Action, EGFR	Tailored to CV risk, comorbidities, and risk modifiers [6]
	Smoking	Patient, GP, caregiver	QUIT smoking	Drug support for smoking cessation (e.g., NRT)	ED, thrombosis, insulin resistance, and dyslipidemia	No smoking
	Dyslipidemia	Patient, GP, caregiver	Diet, exercise	LLT: statins, bempedoic acid, ezetimibe, PCSK9-Is, fibrates	Cholesterol pathway, PCSK9 overexpression in cancer	LLT tailored to CV risk [6] and cancer prevention [152]
	Sedentary behavior	Patient, GP, policymakers (for population-based interventions and the promotion of healthy environments)	Exercise	n/a	Regulation of cardiometabolic and immune function. Oxidative stress, genomic instability, and myokines [6]	Recommended for adults of all ages at least 150–300 min/week of moderate intensity or 75–150 min/w of vigorous intensity aerobic PA. Adults who cannot perform 150 min of moderate-intensity PA/week should stay as active as their abilities and health condition allow [6,152]
	Unhealthy diet, alcohol abuse	Patient, GP, caregiver, institutions (e.g., schools, workplaces, etc.), policymakers	Healthy diet (e.g., mediterranean diet, DASH). Moderate (<100 g/week) or no alcohol [6,152]	n/a.	Oxidative stress	Optimal BMI [2] No alcohol [6,152]
Psycho	**Risk Enhancers**	**Committed Subjects**	**Non-pharmacological** **Management**	**Pharmacological** **Management**	**Pathways**	**Targets**
	Anxiety	Patient, psychological support, caregiver	Healthy lifestyle, psychotherapy, care management approach, group-based cognitive behavioral therapy [2]	Antidepressants (SSRI)	Accelerated development of CV risk factors [176]	Improvement of symptoms, some beneficial effects on CVH
	Anger and hostility	Patient, psychological support, caregiver	Healthy lifestyle		Accelerated development of CV risk factors [176]	Improvement of symptoms, some beneficial effects on CVH
	Pessimism	Patient, psychological support, caregiver	Healthy lifestyle	Psychopharmaco-therapy	Accelerated development of CV risk factors [176]	Optimistic behavior, beneficial effects on CVH
	Depression	Patient, psychological support, caregiver	Healthy lifestyle, psychotherapy, care management approach [2]	Antidepressants (SSRI), psychotherapy, care management approach [2]	Accelerated development of CV risk factors [176], endothelial dysfunction [177,179]	Resolution of symptoms of depression, some beneficial effects on CVH
Exposome	**Social Determinants of Health**	**Committed Subjects**	**Non-pharmacological** **Management**	**Pharmacological** **Management**	**Involved Pathways**	**Targets**
	Environmental conditions: air, noise and light pollution	Policymakers, healthcare practice	Interventions to improve air quality	n/a	Syndemic effect with CV risk factors	Mitigation strategies for air pollution (e.g., transition from fossil fuels to renewable energy sources and transportation reforms), for noise pollution (e.g., implementation of noise reduction protocols, promotion of green spaces as natural sound buffers), and light pollution (energy conservation and light pollution regulations) [57]
	Socioeconomic status	Policymakers, health professionals	Improvement of social conditions	n/a	Stress-associated neural activity), bone marrow activity, and arterial inflammation [175]	Reduction of stressors-induced inflammation, increase in resilience
	Social isolation	Policymakers, health professionals	Tailored interventions	n/a	Increased arterial stiffness [179]	Reduction of loneliness feeling

Abbreviations: ACE-Is: Angiotensin converting enzyme-Inhibitors; AMPK: adenosine monophosphate (AMP)-activated protein kinase; ARBs: Angiotensin Receptor Blockers; BMI: body mass index; CCBs: calcium channel blockers; CRP: C-reactive protein; CV: cardiovascular; CVH: cardiovascular health; DASH: Dietary Approaches to Stop Hypertension; ED: endothelial dysfunction; EGFR: epidermal growth factor receptor; ETC: electron transport chain; GLP-1 RAs: Glucagon-like peptide-1 receptor agonists; GP: general practitioner; IGFR: insulin-like growth factor receptor; LLT: lipid-lowering therapy; MD: Mediterranean diet; MDA: malondialdehyde; mTORC1: mechanistic target of rapamycin complex 1; n/a: not applicable; NO: nitric oxide; NRT: Nicotine Replacement Therapy; PCSK9-Is: proprotein convertase subtilisin/kexin type 9 Inhibitors; SGLT2-Is: sodium-glucose co-transporter-2 inhibitors; SSRI: selective serotonin reuptake inhibitors. n/a: not applicable.

## Abbreviations

ACE-Is	Angiotensin converting enzyme-Inhibitors
ACS	acute coronary syndrome
ADT	androgen deprivation therapy
AHA	American Heart Association
AMI	acute myocardial infarction
AMP	adenosine monophosphate
AMPK	adenosine monophosphate (AMP)-activated protein kinase
ARBs	angiotensin receptor blockers
ASCVD	atherosclerotic cardiovascular disease
ATP	adenosine triphosphate
AYA	adolescent young adult
BMI	body mass index
CAC	coronary artery calcium
CAD	coronary artery disease
CANTOS	Canakinumab Anti-inflammatory Thrombosis Outcome Study
CCB	calcium channel blockers
CCD	chronic coronary disease
CCTA	coronary computed tomography angiography
CHIP	clonal hematopoiesis of indeterminate potential
CKD	chronic kidney disease
CKM	cardiovascular–kidney–metabolic
CKMH	cardiovascular–kidney–metabolic health
CORALS	Childhood Obesity Risk Assessment Longitudinal Study
CRP	C-reactive protein
CT	computed tomography
CV	cardiovascular
CVD	cardiovascular disease
CVH	cardiovascular health
DASH	dietary approaches to Stop Hypertension
DEXA	dual-energy X-ray absorptiometry
ECM	extracellular matrix
ESMO	European society of medical oncology
EVOO	extra virgin olive oil
ETC	electron transport chain
FAI	Fat Attenuation Index
FDA	Food and Drug Administration
FI	Food insecurity
FMF	Familial Mediterranean Fever
GLP-1	glucagon-like peptide 1
GLP-1 RAs	Glucagon-like peptide-1 receptor agonists
HbA1c	hemoglobin A1c
HF	heart failure
HMG-CoA	3-hydroxy-3-methylglutaryl-CoA
hs	high-sensitivity
ICAM-1	intercellular cell adhesion molecule-1
ICB	immune checkpoint blockade
ICIs	immune checkpoint inhibitors
IGFR	insulin-like growth factor receptor
IL	interleukin
IOM	Institute of Medicine
irAEs	immune-related adverse events
LDL-C	low-density lipoprotein cholesterol
LDLRs	LDL receptors
LLT	lipid lowering therapy
LKB1	liver kinase B1
Lp(a)	lipoprotein(a)
LPS	lipopolysaccharide
MACE	major adverse cardiovascular events
MAPK	mitogen-activated protein kinase
MCP-1	monocyte chemoattractant protein-1
MDA	malondialdehyde
MDSCs	myeloid-derived suppressor cells
MESA	Multi-Ethnic Study of Atherosclerosis
MetS	metabolic syndrome
mGPD	mitochondrial glycerol-3-phosphate dehydrogenase
MI	myocardial infarction
MMP-9	matrix metalloproteinase 9
mTORC1	mechanistic target of rapamycin complex 1
NCDs	non-communicable diseases
NET	neutrophil extracellular trap
NF-Kb	nuclear factor-kB
NLRP3	nucleotide oligomerization domain-like receptor protein 3
NLR	neutrophil–lymphocyte ratio
NO	nitric oxide
Non-HDL-C	non-high-density lipoprotein cholesterol
NRT	Nicotine Replacement Therapy
NSCLC	non-small-cell lung cancer
OCT	optical coherence tomography
PA	Physical activity
PCa	prostate cancer
PCEs	Pooled Cohort Equations
PCSK9-Is	proprotein convertase subtilisin/kexin type 9 Inhibitors
PD-L1	programmed death ligand 1
PESA	Progression of Early Subclinical Atherosclerosis
PET	Positron emission tomography
PUFA	polyunsaturated fatty acids
PVAT	perivascular adipose tissue
RCT	randomized controlled trial
SCFAs	short-chain fatty acids
SCORE2	Systematic Coronary Risk Estimation 2
SCORE2-OP	SCORE2-Older Persons Notes
